# Ketone-Based Metabolic Therapy: Is Increased NAD^+^ a Primary Mechanism?

**DOI:** 10.3389/fnmol.2017.00377

**Published:** 2017-11-14

**Authors:** Marwa Elamin, David N. Ruskin, Susan A. Masino, Paola Sacchetti

**Affiliations:** ^1^Neuroscience Program, Department of Biology, University of Hartford, West Hartford, CT, United States; ^2^Neuroscience Program and Psychology Department, Trinity College, Hartford, CT, United States

**Keywords:** ketone bodies, metabolism, hippocampus, epilepsy, neurodegeneration, Alzheimer’s disease, nicotinamide adenine dinucleotide, longevity

## Abstract

The ketogenic diet’s (KD) anticonvulsant effects have been well-documented for nearly a century, including in randomized controlled trials. Some patients become seizure-free and some remain so after diet cessation. Many recent studies have explored its expanded therapeutic potential in diverse neurological disorders, yet no mechanism(s) of action have been established. The diet’s high fat, low carbohydrate composition reduces glucose utilization and promotes the production of ketone bodies. Ketone bodies are a more efficient energy source than glucose and improve mitochondrial function and biogenesis. Cellular energy production depends on the metabolic coenzyme nicotinamide adenine dinucleotide (NAD), a marker for mitochondrial and cellular health. Furthermore, NAD activates downstream signaling pathways (such as the sirtuin enzymes) associated with major benefits such as longevity and reduced inflammation; thus, increasing NAD is a coveted therapeutic endpoint. Based on differential NAD^+^ utilization during glucose- vs. ketone body-based acetyl-CoA generation for entry into the tricarboxylic cycle, we propose that a KD will increase the NAD^+^/NADH ratio. When rats were fed *ad libitum* KD, significant increases in hippocampal NAD^+^/NADH ratio and blood ketone bodies were detected already at 2 days and remained elevated at 3 weeks, indicating an early and persistent metabolic shift. Based on diverse published literature and these initial data we suggest that increased NAD during ketolytic metabolism may be a primary mechanism behind the beneficial effects of this metabolic therapy in a variety of brain disorders and in promoting health and longevity.

## Introduction: Ketogenic Diet and Disorders of The Nervous System

A diet high in fat, low in carbohydrate and sufficient in protein will automatically shift the dependency of energy production in the body from primarily glucose to primarily ketone bodies and is termed a “ketogenic diet” (KD; Branco et al., 2016; Masino, 2017). This dietary approach was developed nearly 100 years ago as metabolic therapy to mimic the metabolic changes that occur during fasting after observing that upon halting food intake, seizures would stop in epileptic people. The KD is well-established as a treatment for epileptic seizures and variations of the diet can be used in children and adults and can be more effective than medication in stopping seizures (Pulford, 1927; Neal et al., 2008). The KD can also prevent seizure progression (epileptogenesis) in animal models and patients (Muller-Schwarze et al., 1999; Neal et al., 2008; Lusardi et al., 2015). Some patients become seizure-free, and remain so even after diet cessation (Martinez et al., 2007; Patel et al., 2010; Caraballo et al., 2011). These lasting outcomes are likely to rely on epigenetic changes (Boison, 2017).

Metabolic dysfunction is increasingly appreciated as a fundamental pathology across disease states (Zhu and Chu, 2010; García-Escudero et al., 2013; Pathak et al., 2013). In models of neurodegenerative diseases, metabolic therapy with a KD or analogous ketone-enhancing metabolic strategies have beneficial effects in cultured neurons, animal models, and in patients. The ketone body β-hydroxybutyrate (β-OHB) protected cultured dopaminergic substantia nigra cells from N-methyl-4-phenylpyridinium (MPP^+^) toxicity and hippocampal neurons from amyloid β toxicity (Kashiwaya et al., 2000), and improved the disease rating score in Parkinsonian patients (Vanitallie et al., 2005). *In vivo* and *in vitro* administration of ketone esters reduced histological and biochemical pathologies and improved cognition, anxiety and motor performance in mouse models of Alzheimer’s disease (Liu et al., 2011; Hui et al., 2012; Brownlow et al., 2013; Kashiwaya et al., 2013; Zhang et al., 2013; Pawlosky et al., 2017). KD improved memory of patients with mild cognitive impairment (Krikorian et al., 2012), and administration of a ketone ester or medium chain triglycerides (often a component of ketogenic treatment) enhanced memory and cognition in Alzheimer’s patients (Reger et al., 2004; Newport et al., 2015; Cunnane et al., 2016). Treatment with a KD suppressed inflammation and improved motor disabilities in a multiple sclerosis model (Kim et al., 2012), altered disease progression and improved motor performance and neuronal survival in an amyotrophic lateral sclerosis model (Zhao et al., 2006), decreased the expression of apoptotic mediators in a traumatic brain injury model (Hu et al., 2009), and improved motor outcomes in a spinal cord injury model (Streijger et al., 2013).

It is becoming apparent that beneficial effects of ketogenic therapy extend beyond epilepsy, neurodegenerative disorders, and brain/spinal cord injury. The KD is broadly effective in improving core behavioral symptoms in animal models of autism spectrum disorder (Ruskin et al., 2013b, 2017a,b; Ahn et al., 2014; Verpeut et al., 2016; Castro et al., 2017; Dai et al., 2017), and in autistic patients (Evangeliou et al., 2003; Masino et al., 2011b; Spilioti et al., 2013). The KD is receiving growing interest in oncology as tumors are highly glucose-dependent (the Warburg effect; Seyfried and Mukherjee, 2005; Zuccoli et al., 2010; Schmidt et al., 2011; Klement et al., 2016; Lussier et al., 2016; Khodadadi et al., 2017). Also, due to the high efficiency of metabolizing fat when carbohydrates are minimal (Forsythe et al., 2010), the KD has been promoted for weight reduction (Jenkins et al., 2009; Partsalaki et al., 2012; Paoli, 2014; Gomez-Arbelaez et al., 2017) and for treatment or reversal of type II diabetes and metabolic syndrome (Yancy et al., 2005; Volek et al., 2008, 2009; Westman et al., 2008; Hussain et al., 2012; Tay et al., 2015; McKenzie et al., 2017).

In addition, healthy, disease-free cells and animals can also benefit from this therapy. The use of ketone bodies as an energy source appears to be associated with a healthier metabolic phenotype that renders cells more resistant to external insults. Ketogenic treatment decreased myocardial damage after ischemic injury, reduced lung injury after hemorrhagic shock, enhanced kidney resistance to oxidative stress, and protected neurons against glutamate-induced toxicity (Zou et al., 2002; Koustova et al., 2003; Noh et al., 2006; Shimazu et al., 2013). At the cognitive level, beneficial effects on learning and memory were reported (Brownlow et al., 2017; Newman et al., 2017). KD in mice started at 8 weeks of age did not affect longevity (Douris et al., 2015); however, KD started midlife extends longevity and healthspan (Newman et al., 2017; Roberts et al., 2017).

## Mechanisms of Ketogenic Therapy: Evidence for Increased NAD^+^

Many mechanisms have been proposed to explain the anti-seizure and neuroprotective effects of the diet, such as enhanced mitochondrial biogenesis (Bough et al., 2006), decreased formation of reactive oxygen radicals (Sullivan et al., 2004), altered transmitter levels and ion channel function (Schwartzkroin, 1999; Bough and Rho, 2007), increased adenosine (Masino et al., 2011a; Masino and Rho, 2012), and decreased DNA methylation (Kobow et al., 2013; Lusardi et al., 2015). Each one of these mechanisms could account for some of the beneficial effects of the ketogenic therapy. However, to date fundamental metabolic mechanism(s) which could explain diverse beneficial effects across numerous diseases have yet to be confirmed. If uncovered, such mechanism(s) could provide a fundamental answer to “how does the KD work?”—a lingering question and a topic of intense resurgent research efforts and clinical interest. A unifying mechanism of action could also serve as a target for the development of therapeutics that enhance cellular and metabolic health and provide the metabolic resilience necessary to prevent and combat neurological diseases.

**Figure 1:**
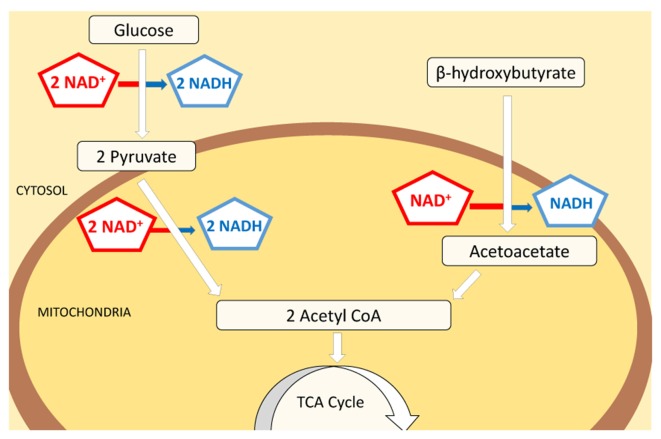
Schematic of NAD^+^ consumption during metabolism of glucose vs. ketone bodies. Both glucose and ketone bodies lead to the formation of two molecules of acetyl-CoA which subsequently enter the citric acid cycle and participate in energy production. Although glucose provides a higher final yield of ATP, the consumption of NAD^+^ is significantly higher in this pathway (4:1). Glucose will reduce 111 molecules of NAD^+^ per 1000 molecules of ATP made, while ketone bodies reduce only 41 to produce a comparable amount of ATP. Decreased use of NAD^+^ by ketone bodies in energy production pathways could increase the amount of free NAD^+^ available as substrate for enzymes and cellular signaling processes.

Glucose and ketone bodies are used to provide energy in the form of ATP. Many tissues in the body—such as muscle tissues—can oxidize fatty acids to produce energy. As one exception, in the central nervous system ketolysis is expected to be the primary pathway of energy production: neurons and oligodendrocytes have a limited capacity for mitochondrial fatty acid β oxidation (Edmond, 1992; Achanta and Rae, 2017). The ketone bodies acetoacetate (AcAc) and β-OHB are therefore the main energy source in the brain during ketosis, and the metabolism of glucose versus ketone bodies results in a differential reduction rate of nicotinamide adenine dinucleotide (NAD), an essential metabolic coenzyme and signaling molecule. NAD exists in oxidized and reduced forms, NAD^+^ and NADH, respectively, and whereas both glucose and ketone pathways each produce two molecules of acetyl-CoA, glucose reduces four molecules of NAD^+^ and ketone bodies reduce either one (β-OHB), or none (AcAc) during acetyl-CoA synthesis (Lodish et al., 2000; Cotter et al., 2013; Figure 1). A decreased reduction of NAD^+^ in the brain would be expected to result in increased NAD^+^/NADH ratio, with more oxidized molecules available for bioenergetic demands.

Increasing the NAD^+^/NADH ratio has multiple important implications: improved bioavailability of NAD^+^ molecules has been linked to anti-aging (Scheibye-Knudsen et al., 2014), longevity (van der Veer et al., 2007; Zhang et al., 2016) and other potentially beneficial effects. For example, an increased NAD^+^/NADH ratio was found to enhance mitochondrial function and protect against oxidative stress, and diverse research has shown that NAD molecules play an important role in cellular respiration, mitochondrial biogenesis and redox reactions (Yang and Sauve, 2016). NAD^+^ also serves as substrate for enzymes affecting cellular functions ranging from gene expression to post-translational protein modifications, such as deacetylation and ADP-ribosylation (Belenky et al., 2007).

We propose that the decreased reduction rate of NAD^+^ to NADH during ketone-based metabolism increases availability of NAD^+^ and thus alters the NAD^+^/NADH ratio. This would occur during sufficient exogenous ketone administration or during fasting or adhering to a KD, i.e., when ketones are used as a main source of energy. Considering the pivotal role of NAD^+^ in cellular health, and that differential NAD reduction is inherent in this metabolic pathway, we suggest that this differential rate of NAD^+^ reduction (and thus an increase in NAD^+^ availability) is a primary mechanism of ketogenic therapy: increased NAD^+^ can potentially be the starting point for many of the diverse benefits of this metabolic treatment.

## Ketogenic Diet-Induced Increase in Brain NAD^+^/NADH Ratio Is Rapid, Persistent and Region Specific

**Figure 2:**
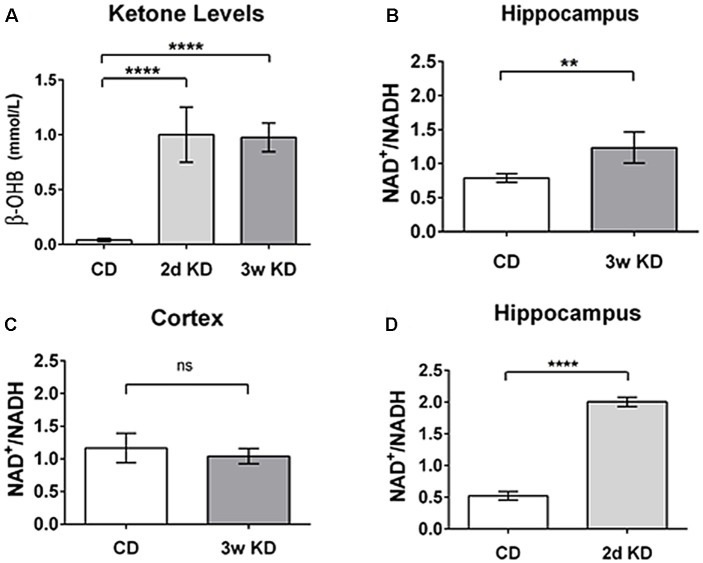
Changes in blood ketones and brain nicotinamide adenine dinucleotide (NAD) after ketogenic diet (KD) treatment.** (A)** Blood levels of β-hydroxybutyrate (β-OHB; mmol/L) after 2 days (2d KD; *n* = 3) and 3 weeks (3w KD; *n* = 4) of KD treatment vs. control chow diet (CD; *n* = 8; *P* < 0.0001). **(B)** Hippocampal changes in NAD^+^/NADH ratio after 3 weeks KD treatment. A significant increase in the NAD^+^/NADH ratio was quantified in the hippocampi of animals fed KD for 3 weeks (*n* = 4) vs. animals maintained on control diet (*n* = 8; *P* < 0.005). **(C)** Cortical NAD^+^/NADH ratio after 3 weeks KD treatment. No differences were detected in NAD^+^/NADH ratio in frontal cortex between the dietary groups. Control CD (*n* = 8); KD 3 weeks (3w KD; *n* = 4; *P* = ns). **(D)** NAD^+^/NADH ratios in the hippocampus after 2 days KD treatment. A significant increase in the NAD^+^/NADH ratio was quantified in hippocampi obtained from animals fed KD for 2 days (2d KD; *n* = 3) compared to animals maintained on control diet (CD; *n* = 5; *P* < 0.0001). All comparisons were unpaired *t*-tests. Data are expressed as mean ± SEM. **P < 0.005; ****P < 0.0001.

As an initial test of this hypothesis, we quantified and compared KD-induced changes in blood ketones and in NAD^+^/NADH ratio in hippocampus and cerebral cortex of normal adult rats. Sprague-Dawley male rats (Trinity College, age 9–14 weeks, *n* = 20) were fed *ad libitum* either a standard chow diet (CD; Purina 5001; PharmaServ, Framingham, MA, USA), or a 6:1 [fat: (protein + carbohydrates); #F3666; Bio-Serv, Frenchtown, NJ, USA] KD for 2 days or 3 weeks. Analysis of trunk serum collected showed that a KD induced a significant increase in ketone bodies (β-OHB; the primary circulating ketone body; Figure 2A), consistent with previous experimental and clinical work (Nabbout et al., 2010; Ruskin et al., 2013a).

To begin to evaluate if, when and where alterations in NAD occurred in brain during ketone-based metabolism we performed NAD analysis on two regions, hippocampus and frontal cortex, after either a 2 days or a 3 weeks KD treatment. Three weeks exposure has been demonstrated to impact behavior and neuronal excitability in diverse paradigms (Hori et al., 1997; Cullingford et al., 2002; Ruskin et al., 2009; Masino et al., 2011a). As demonstrated by ketone body levels (Figure 2A), metabolic changes were significant within 2 days.

Twenty milligram samples of tissue from each region were homogenized in NAD extraction buffer, centrifuged and deproteinized using 10 kDa molecular cut-off filters. Analysis of NAD was performed using an enzymatic NAD^+^/NADH quantification kit (Sigma Aldrich, St. Louis, MO, USA) according to manufacturer’s instructions. Fractions of samples were incubated for 5 min at room temperature for the detection of total NAD (tNAD), while equal amounts were heated to 60°C for 30 min to decompose NAD^+^ and leave unaltered NADH. tNAD and NADH were quantified with a colorimetric measure. Values of NADH were subtracted from tNAD to calculate the amount of NAD^+^ present in the samples.

After 3 weeks of treatment, extracted hippocampi showed a significant increase in NAD^+^/NADH ratio in the KD-fed group compared to the CD-fed group (Figure 2B). Interestingly, no detectable changes in NAD^+^/NADH ratios were observed in frontal cortices of the same animals (Figure 2C).

Interestingly, a robust and similar increase in NAD^+^/NADH ratio was already detectable in hippocampi after 2 days of KD treatment (Figure 2D). This result aligns with previous work (Nabbout et al., 2010; Ruskin et al., 2013a) showing that metabolic changes are present within 2 days and further corroborates the evidence that ketone bodies increase NAD^+^ availability rapidly. Increases in NAD^+^/NADH ratios were due to increases in NAD^+^ as NADH levels were not changed significantly by diet treatments (CD: 3.25 ± 0.32 pmol/μg proteins; 2 days: 2.58 ± 0.21; 3 weeks: 2.68 ± 0.21; not significant). The changes quantified in the hippocampus are consistent with the predicted metabolic consumption, as highlighted in the metabolic pathways (Figure 1).

## Perspective and Implications

Consistent with our predictions we found clear evidence that metabolic therapy with a KD increases NAD^+^/NADH, a mechanism that could compensate for metabolic dysregulation and serve as a common start-point for the diverse beneficial metabolic and mitochondrial effects obtained with ketogenic treatments (Bough and Rho, 2007; Masino and Geiger, 2008). As noted above, a comparison of the metabolic pathways of glucose and ketone bodies (Figure 1) suggests that the use of ketone bodies as main energy fuel requires fewer NAD^+^ molecules than glucose (by a factor of 4), which should lead to an increased cellular availability of this vital coenzyme. Interestingly, β-OHB or a ketone ester precursor show protective effects by counterbalancing the decrease in NAD^+^/NADH ratio in cases of neurotoxicity (Maalouf et al., 2007; Zhang et al., 2013), confirming the ability of β-OHB to modulate NAD^+^/NADH levels. Ketone ester treatment oxidizes the cytoplasmic NAD^+^/NADH couple in hippocampus and cortex in aged, affected Alzheimer’s disease model mice, and reverses an apparent overoxidation of the mitochondrial couple in the hippocampus (Pawlosky et al., 2017). Thus this study and our study of the whole-cell couple suggest clear positive metabolic effects of ketolytic metabolism.

In general, the hippocampus has been described as a seizure gate (Heinemann et al., 1992) and it is one of the first brain regions to be affected in Alzheimer’s type dementia; cortical changes appear later in the disease (Braak and Braak, 1998). Unexpectedly, our data show that a KD increased NAD^+^/NADH in the hippocampus but not in the cerebral cortex, indicating regional specificity at the time points sampled. It is possible that changes mobilized by a KD could be more rapid or pronounced in more metabolically active brain regions: the hippocampus displays a higher metabolic rate than the cerebral cortex (Feng et al., 1988). Related to this, an early decrease in metabolic rate of glucose in the hippocampus, but not in cerebral cortex, was detected in patients who received a postmortem diagnosis of Alzheimer’s disease (Mosconi et al., 2009). Because of its high metabolic rate and increased vulnerability to hypoxia, oxidative stress, and metabolic dysfunction, the hippocampus could benefit more—or more rapidly—than other regions from KD-induced metabolic changes, including increased NAD^+^ levels.

Rapid increases in NAD^+^/NADH ratio could partially explain the ability of a KD to stop seizures in many patients within a few days of KD treatment (Freeman and Vining, 1999). For example, NAD^+^ and NADH molecules can modulate directly the opening of ion channels important for neuronal excitability, such as ATP-sensitive and voltage-gated potassium channels (Dukes et al., 1994; Tipparaju et al., 2005). Accordingly, a rapid decrease in NAD^+^ availability and consequent effect on neuronal excitability should be expected upon discontinuation of treatment. Interestingly, 15% of refractory epileptic patients experienced a rapid recurrence of seizures after KD discontinuation (Martinez et al., 2007); others remain seizure-free. The differential response among patients to treatment cessation indicates the existence of multiple downstream mechanisms and epigenetic changes (Masino and Rho, 2012; Lusardi et al., 2015) implicated in seizure control. Upregulation of key ketogenic enzymes, mainly mitochondrial 3-hydroxy-3-methylglutaryl-CoA synthase, after longer periods of ketogenic treatment (Cullingford et al., 2002) might also play a role in the maintenance of the beneficial effects even after discontinuation of the diet. More work is needed on downstream and lasting effects of metabolic therapy.

Consistent with these reported effects, previous work showed that addition of ketone bodies also prevented the expected decrease in NAD^+^/NADH ratio induced by toxic doses of calcium and in parallel decreased the production of reactive oxygen species (ROS), a major source of cellular oxidative stress (Maalouf et al., 2007); oxidative stress is detrimental and linked to neuronal death and neurodegenerative diseases (Naoi et al., 2005). Altering the NAD^+^/NADH ratio can control the rate of ROS production (Kussmaul and Hirst, 2006) and impact downstream enzymatic levels and activities that regulate apoptosis and inflammation (Yeung et al., 2004; Chen et al., 2008; Zhu et al., 2011). Enhanced NAD^+^/NADH should thus decrease inflammation, an effect observed in KD treatment (Forsythe et al., 2008; Ruskin et al., 2009; Yang and Cheng, 2010; Dupuis et al., 2015; Nandivada et al., 2016). Overall, increasing the NAD^+^/NADH ratio through a number of ketone-enhancing treatments should protect against oxidative stress and enhance mitochondrial and cellular health.

Regarding gene expression, consuming a KD has been found to impact expression patterns of genes modulating neuroinflammation, proliferation, and apoptosis such as cyclooxygenase, tumor necrosis factor-α, and insulin-like growth factor 1 (Cheng et al., 2003; Jeong et al., 2011). Although increased NAD^+^ cannot exert these effects directly, NAD^+^ can impact gene expression through the action of sirtuin enzymes. Increasing NAD^+^ availability or the NAD^+^/NADH ratio can increase the activity of the NAD-dependent SIRT1 enzyme (the most abundant member of the sirtuin family; Landry et al., 2000; Chen et al., 2008). The main function of SIRT1 is deacetylation of targets that regulate apoptosis and transcription factors such as peroxisome proliferator-activated receptor-γ and the tumor suppressor protein p53 (Yeung et al., 2004; Zhu et al., 2011). Hence, some of the expected downstream consequences of increasing NAD^+^ with ketogenic treatment are decreased cell death, inhibition of inflammation, and modulation of gene expression and epigenetic changes through activation of sirtuin enzymes (Janke et al., 2015).

## Conclusion

Here we outline the overall implications and evidence for a rapid and region-specific change in NAD^+^/NADH as a direct result of consuming a KD. We hypothesize this as a new and fundamental addition to potential key mechanisms underlying beneficial antiseizure, neuroprotective and disease-modifying effects of KD. Because NAD^+^ can modulate ion channels, enhance mitochondrial health, decrease oxidative stress, and impact gene expression, an increase in NAD^+^ and/or NAD^+^/NADH ratio is a mechanism that can account for several diverse (and seemingly-unrelated) effects of ketogenic therapy. Furthermore, benefits of increasing NAD^+^ such as life-span extension and enhancing cellular health have long been documented (Lin et al., 2000), and pharmaceutical companies are currently manufacturing and selling supplements that contain NAD^+^ precursors such as nicotinamide or nicotinamide riboside in an attempt to increase endogenous NAD^+^ levels and enhance metabolic resilience—an outcome that may also be achieved physiologically by ketogenic strategies.

At this time more research is needed to further identify where and when ketogenic therapy increases the NAD^+^/NADH ratio, and to delineate specific downstream effects. It is also important to ascertain if changes in the NAD^+^/NADH ratio are caused by changes in NAD^+^ or NADH, as levels of these two redox molecules can also vary independently (Wilhelm and Hirrlinger, 2011). Regardless, diverse lines of evidence place NAD^+^ at the center of metabolic health and disease, and evidence from our work and others supports the hypothesis that increased NAD^+^ is a fundamental molecular mechanism of the KD. Our findings also indicate the potential for a greater role for this metabolic therapy in areas with high metabolic demand and vulnerability to environmental insults and oxidative stress such as the hippocampus. Taken together, increasing brain NAD^+^ levels—either by consuming a KD or by other ketone-enhancing treatments—might serve as a rapid and enduring strategy to halt or even reverse disease progression.

## Ethics Statement

This study was carried out in accordance with the recommendations of National Institutes of Health (NIH) Guides and Trinity College Animal Care and Use Committee. The protocol was approved by the Trinity College Animal Care and Use Committee.

## Author Contributions

ME, DNR, SAM and PS: conception and design of experiments; analysis and/or interpretation of data; drafting the manuscript; revising the manuscript critically for important intellectual content and approval of the version of the manuscript to be published. ME and DNR: acquisition of data.

## Conflict of Interest Statement

The authors declare that the research was conducted in the absence of any commercial or financial relationships that could be construed as a potential conflict of interest.
